# From grass to legume: employing *Poa pratensis*-associated bacteria to improve soybean performance under salinity stress

**DOI:** 10.3389/fpls.2026.1748426

**Published:** 2026-04-01

**Authors:** Nadia Monjezi, Hamid Reza Eisvand, Sima Abdoli, Donald L. Smith

**Affiliations:** Plant Science Department, McGill University, Montreal, QC, Canada

**Keywords:** abiotic stress, bio-inoculant, endophytic bacteria, phytohormone, plant growth-promoting bacteria (PGPB)

## Abstract

Soil salinity is one of the major abiotic stresses limiting agricultural productivity worldwide, and strategies to enhance plant tolerance are of critical importance. Soybean (*Glycine max*) is a globally important legume crop, providing a vital source of protein for human consumption and livestock feed, as well as serving as a raw material for various industrial products. In this study, endophytic bacteria were isolated under two temperatures (5 °C and 28 °C) in TYE media from the roots of *Poa pratensis*. Preliminary screening for plant growth-promoting activity was performed using a soybean germination assay under salt stress conditions (0, 75, 100 mM NaCl). Among the isolates, two strains showing the most pronounced positive effects on germination rate and percentage were selected for molecular identification. Phylogenetic analysis of 16S rRNA gene sequences with bootstrap support demonstrated that both isolated strains belong to the genus *Pseudomonas*. The growth of these strains was monitored in saline media and demonstrated tolerance up to 500 mM NaCl. Biochemical characterization showed their ability to produce auxins, siderophores, and solubilize phosphate. In pot experiments, soybean seeds were inoculated with these strains, and salinity stress was applied 24 hours after inoculation. The results demonstrated that the inoculated plants exhibited improved growth under salt stress, including enhanced root development, greater dry biomass accumulation, and increased overall vigor. These findings suggest that *Pseudomonas* endophytes isolated from *Poa pratensis* possess potential, as bio-inoculants, to mitigate the adverse effects of salinity and promote sustainable soybean cultivation.

## Introduction

In the face of climate change, there is a consensus across scientific and industrial sectors about the urgency of finding solutions to mitigate its impacts. In agriculture, these efforts are particularly critical, as food production must keep pace with a rapidly growing global population. However, climate change and intensive agricultural practices, particularly the excessive use of chemical fertilizers, are mutually reinforcing. Climate change imposes greater stress on crops through increased frequency of extreme weather events, while intensive fertilizer use contributes to climate change through greenhouse gas emissions during production and through environmental release after field application, through leaching into groundwater, running off into rivers and lakes, or release as gases into the atmosphere, all of which harm ecosystems and make climate problems worse (Liu et al., 2025; Jang et al., 2025; Yang et al., 2024).

Among the various abiotic stresses intensified by these factors, soil salinity stands out as a major constraint limiting crop yields worldwide, especially in arid and semi-arid regions. The global increase in soil salinization is driven largely by poor irrigation practices coupled with climate change impacts. Changes in precipitation patterns and seawater intrusion into coastal farmlands further exacerbate salt stress on crops. In addition, the over-application of fertilizers can worsen soil salinity by increasing the accumulation of soluble salts in the root zone. Excessive fertilizer application also contributes to chemical buildup in soils, such as nitrates and phosphates, which can degrade soil health and lead to water pollution (Palmgren and Shabala, 2024; Zhang et al., 2015).

Sustainable alternatives are crucial for addressing the harmful impacts of excessive fertilizer application. Among these, biostimulants present a promising strategy by boosting plants’ capacity to access and absorb nutrients already present in the soil, thereby lowering dependence on synthetic fertilizers (Du Jardin, 2015). They also contribute to greater plant tolerance to climate-change-related stress by promoting overall health and activating natural defense systems (Rouphael and Colla, 2020). Various types of biostimulants, such as microbial inoculants, seaweed extracts, and humic substances, can encourage root growth and enhance soil microbial activity, ultimately improving nutrient availability (Calvo et al., 2014). Furthermore, by increasing nutrient use efficiency, biostimulants can help to maintain crop productivity under challenging conditions. Their ability to stimulate antioxidant responses, regulate osmotic balance, and influence stress-related gene expression helps plants better endure abiotic stress factors like salinity, heat, and drought (Yakhin et al., 2017; Rouphael et al., 2018).

Plant Growth-Promoting Rhizobacteria (PGPR) are increasingly recognized as a sustainable alternative to chemical fertilizers and pesticides. These beneficial microbes, commonly found in the rhizosphere, support plant growth by enhancing nutrient uptake, producing growth-regulating hormones, and helping plants withstand abiotic stressors such as drought and salinity (Lugtenberg and Kamilova, 2009). Wild plants, which are often better adapted to fluctuating environmental conditions, harbor diverse and resilient microbial communities. These microbes represent a rich source of genetic and functional diversity that can be harnessed to improve the resilience of domesticated crops, which typically have reduced adaptability to environmental stress due to selective breeding (Fitzpatrick et al., 2018). Unlike crops, wild plant species have evolved in complex and dynamic ecosystems, forming robust associations with PGPR that enable them to thrive in nutrient-poor or saline soils, under heat or drought stress, and without human intervention (Berg et al., 2017; Escudero-Martinez and Bulgarelli, 2023).

The concept of “wild-to-crop microbiome transfer” has therefore gained attention as a strategy to introduce environmentally adapted beneficial microbes into agricultural systems (Sessitsch et al., 2019). However, most studies investigating PGPR-mediated salinity tolerance in soybean have focused on strains isolated from conventional agricultural soils. Comparatively less attention has been given to microorganisms originating from ecologically dynamic habitats exposed to natural salinity fluctuations.

In this context, the present study explores salt-tolerant PGPR isolated from the rhizosphere of *Poa pratensis* growing in a shoreline habitat characterized by periodic salinity exposure. We hypothesize that bacteria originating from such environments possess adaptive functional traits—such as enhanced salt tolerance, osmotic regulation capacity, and stress-responsive metabolite production—that distinguish them from previously reported strains. By evaluating their effects on soybean (*Glycine max*) germination and growth under saline conditions, this study aims to provide new insights into the ecological significance of habitat-driven microbial adaptation and its application in sustainable crop improvement.

## Materials and methods

### Sample collection and bacterial isolation

Plants of the species *Poa pratensis* were collected from the natural vegetation along the shore of Lac St. Louis on the Macdonald Campus of McGill University, located in Sainte-Anne-de-Bellevue, Quebec, Canada. Roots were separated from the shoots, thoroughly washed, and surface sterilized with 70% (v/v) ethanol for 2 minutes. After sterilization, the roots were rinsed three times with sterile distilled water. To confirm the effectiveness of surface sterilization, the final rinse water was plated on nutrient agar and no bacterial growth was observed. So, the isolates obtained were considered endophytic. The sterilized roots were crushed using sterile micro pestles, and the resulting suspension was serially diluted in sterile distilled water. Dilutions ranging from 10^-^² to 10^-7^ were plated onto Tryptone Yeast Extract (TYE) agar. Plates were incubated at both 28 and 4 °C for 24 to 96 hours. Morphologically distinct bacterial colonies (excluding molds and actinomycetes) were selected and re-streaked on fresh TYE agar plates to obtain pure cultures. Eight isolates were cultivated in liquid broth and preserved in 25% glycerol stocks at −80 °C for long-term storage.

### Preparation of bacterial cultures

Bacterial strains were cultured in Tryptone Yeast Extract (TYE) broth for 48 hours at 28 °C with shaking at 150 rpm. Cells were harvested by centrifugation at 10, 000 × g for 20 minutes at 5 °C using an Awel™ MF 48-R centrifuge (NuAire, USA). The supernatant was discarded, and the resulting cell pellet was resuspended in 10 mM MgSO_4_, and the optical density was adjusted to the desired OD, depending on the experiment, at 600 nm using an Ultraspec 4300 pro UV/Visible Spectrophotometer (Biochrom). For example, bacterial suspensions with OD values of 0.001, 0.01, and 0.1, corresponding to approximately 10^6^, 10^7^, and 10^8^ CFU mL^-1^, respectively, were subsequently used for the germination experiments and greenhouse pot experiments.

### Identification of isolated bacteria

The bacterial isolates were identified through Sanger sequencing of the 16S rRNA gene, conducted by Genome Quebec (Montreal, Canada). Prior to amplification, DNA samples were diluted 1:10 with sterile water. PCR reactions were prepared using Taq DNA polymerase (Roche FastStart High Fidelity PCR System, 2500 U) along with the universal bacterial primers 27F (5′-AGAGTTTGATCMTGGCTCAG-3′) and 1492R (3′-TACGGYTACCTTGTTACGACTT-5′). Amplification was carried out using an Eppendorf Mastercycler^®^ ProS thermal cycler for 40 cycles. The PCR products were sequenced using the Applied Biosystems™ 3730XL DNA Analyzer.

The resulting forward sequence reads (FASTA format) were analyzed for similarity using the BLAST algorithm against reference prokaryotic type strains available in the NCBI database (https://blast.ncbi.nlm.nih.gov/). Taxonomic identification at the genus level was based on alignment scores and percent identity. The 16S rRNA gene sequences of *Pseudomonas* strains with complete genome sequences were retrieved from the NCBI database. Sequence alignment and phylogenetic tree construction were performed using MEGA version 12. The evolutionary history was inferred using the Neighbor-Joining method, and the reliability of the tree topology was evaluated by bootstrap analysis with 1000 replications.

### Germination experiments

Soybean untreated seeds (B103EE) were surface sterilized in 5% sodium hypochlorite for 1 minute and rinsed three times with sterile distilled water. Ten sterilized seeds were placed on filter paper in each Petri dish and treated with 5 mL of bacterial suspensions at different concentrations (10^6^, 10^7^, and 10^8^ CFU mL^-1^) prepared in sterile distilled water containing MgSO_4_ 10 mM. For salt stress treatments, NaCl was added to the bacterial suspensions at 75 or 100 mM, while MgSO_4_ 10 mM was maintained in all solutions. Control groups received MgSO_4_ 10 mM only, with or without NaCl at 75 or 100 mM, to account for the effects of MgSO_4_ and salt independently of bacterial treatment. Petri dishes were sealed in polyethylene bags to reduce evaporation and incubated in a growth chamber at 25 ± 2 °C, 70% relative humidity, in darkness. Germination was monitored several times each day for 72 hours. Seeds were considered germinated when the radicle exceeded 0.2 cm in length. The number of germinated were recorded for each treatment. Germination rate (GR) was calculated using the formula below (Maguire, 1962):

GR (seed day^-1^) = (n_1_/d_1_) + (n_2_/d_2_) +… + (n_k_/d_k_), where “n” is the number of germinated seeds and “d” is the time in days.

### Greenhouse experiments

Untreated seeds (five per pot) were sown in 15.25 cm diameter pots filled with pre-irrigated vermiculite (Perlite Canada Inc Each seed was inoculated with 500 µL of bacterial suspension containing 10^6^, 10^7^, or 10^8^ CFU mL^-^¹ before being covered with vermiculite. At the time of sowing, pots had already received 300 mL of water and were moist.

One day after seeding, pots were irrigated with either 300 mL of distilled water (non-saline control) or NaCl solutions at 75 mM, 100 mM, and 125 mM. Thereafter, all treatments were irrigated with equal volumes of tap water (100–120 mL) as needed, ensuring that no leaching occurred from the pots. Pots were maintained under greenhouse conditions at 25 ± 2 °C and 70% relative humidity. Seedlings were thinned to one plant per pot at 7 days after planting (DAP). Plants were irrigated daily with 100 mL of water and received weekly supplementation with Hoagland’s nutrient solution.

Chlorophyll index was measured at 26 days after planting (DAP) using a SPAD-502 chlorophyll meter (Konica Minolta Sensing, Inc., Japan). At 28 DAP, plants were harvested for growth analysis, which included measurements of plant height, leaf area, shoot dry weight, and root dry weight. Root systems were scanned using an EPSON Expression 11000XL scanner and analyzed with WinRHIZO™ software (Regent Instruments Inc., Quebec, Canada) to quantify root volume, total root length, and number of root tips.

### Characterization of selected bacterial isolates for plant growth-promoting traits

#### Indole-3-acetic acid production, siderophore production, and phosphate solubilization tests

The production of indole-3-acetic acid (IAA) was quantified following the procedure outlined by Naik and Sakthivel (2006). Siderophore synthesis by the bacterial isolates was assessed using the Chrome Azurol S (CAS) assay as described by Alexander and Zuberer (1991). For the assessment of phosphorus solubilization, bacterial strains were inoculated on Pikovskaya agar medium (Pikovskaya, 1948). Thus, the appearance of a clear halo surrounding the bacterial colonies was considered indicative of phosphate solubilization activity.

#### Nitrogen fixation ability

The nitrogen fixation ability of bacterial isolates was evaluated using the NFb semi-solid medium described by Baldani et al. (2014). This medium contains malic acid as the primary carbon source, along with essential macro- and micronutrients, vitamins, and the pH indicator bromothymol blue. The pH was adjusted to 6.5, and 1.8 g L-1 of agar was added to prepare the semi-solid medium. Bacterial isolates were inoculated into the semi-solid NFb medium and incubated at 30 °C for 3–5 days. Nitrogen-fixing activity was evidenced by the formation of a subsurface pellicle under microaerophilic conditions.

#### Salinity tolerance

Salt tolerance of the bacterial isolates was assessed using a microplate reader (Cytation 5, BioTek, USA) with Gen5 software. A 96-well microplate was prepared, with each well containing 200 µL of culture composed of 20 µL bacterial inoculum (OD600 = 0.1) and 180 µL TYE medium supplemented with different NaCl concentrations. The plates were incubated at 28 °C for 48 h, and optical density was automatically recorded at 2 h intervals. All treatments were performed with four biological replicates.

#### Data analysis

Minitab 22 software was used to analyze the data and draw the graphs. For the germination assays, independent t-tests were applied during the initial screening to compare each superior treatment with its corresponding control within the same salinity level. Following the identification of the two superior strains, additional germination test was done and data for these strains were further analyzed using one-way ANOVA, with Tukey’s HSD test used for multiple comparisons among CFU levels. The greenhouse experiment was conducted twice. Prior to combined analysis, homogeneity of variances was tested. For root length, variances were not homogeneous between experiments; therefore, data from the two experiments were analyzed separately.

## Results

### Identification of isolated bacteria and germination experiments

A total of eight bacterial strains were isolated from the root of *Poa pratensis* grown on TYE medium. Among these, three strains were isolated at 28 °C and five strains at 5 °C. All isolates were evaluated in a seed germination assay to assess their potential to enhance plant tolerance under salt stress. The results indicated that strains 5 and 7 exhibited the best performance in mitigating the negative effects of salinity. Consequently, 16S rRNA gene sequencing was performed for these two superior isolates to determine their taxonomic affiliation.

### 16S rRNA gene sequencing analysis

The 16S rRNA gene sequences of the isolates were successfully determined and compared with those available in the NCBI GenBank database using BLAST. Both isolates showed the highest similarity to members of the genus *Pseudomonas*. The sequence similarity values were 99.74 and 100% for the two isolates, indicating a close relationship with *Pseudomonas* species. Detailed information, including the closest related species, percentage identity, query coverage, and GenBank accession numbers, is provided in **Table 1**.

**Table 1:** Closest relatives of bacterial isolates based on 16S rRNA gene sequence analysis.

Isolate ID and gene bank accession no.	Closest species (NCBI BLAST)	PCR amplicon size (bp)	Query coverage (%)	Identity (%)	Gene bank accession no.
Isolate 5 SLNM5 PX462032	*Pseudomonas lyxosi* LB3P38* 16S ribosomal RNA, partial sequence	783	100	99.74	NR_200030
Isolate 7 SLNM7 PX462032	*Pseudomonas mandelii* CIP 105273 16S ribosomal RNA, partial sequence	806	100	100	NR_024902

*Species names represent closest BLAST matches based on partial 16S rRNA sequences and do not indicate definitive species-level identification.

**Figure 1:**
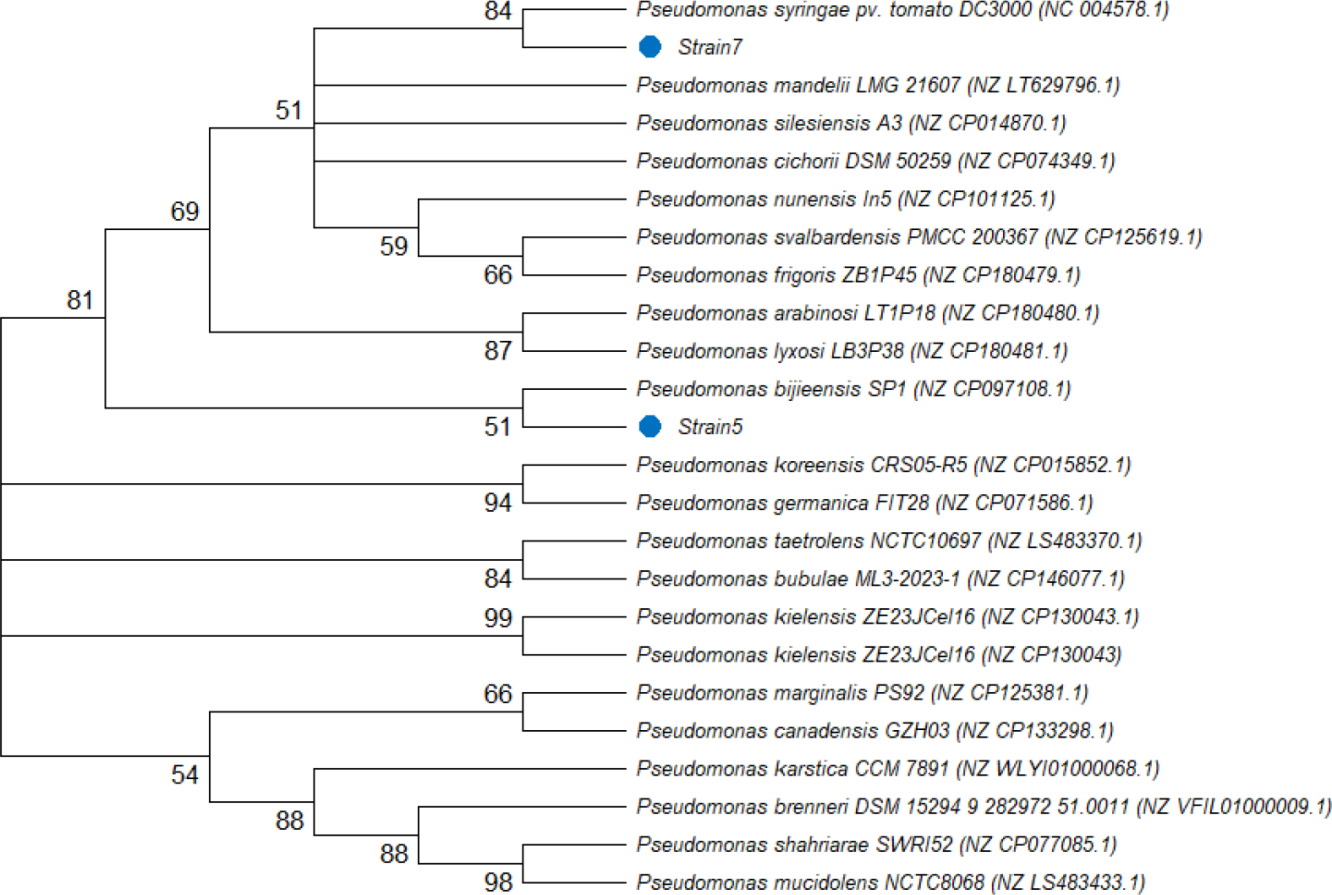
Neighbor-joining phylogenetic tree based on 16S rRNA gene sequences showing the relationship between the studied strains and reference *Pseudomonas* species. Bootstrap values are indicated at branch nodes.

Phylogenetic analysis based on 16S rRNA gene sequences placed both isolated strains within the *Pseudomonas* cluster (**Figure 1**). Strain7 grouped closely with *Pseudomonas syringae* pv. *tomato* DC3000, *P. mandelii*, and *P. silesiensis*, with a bootstrap value of 84, indicating a well-supported relationship within this clade. Strain5 formed a sister relationship with *Pseudomonas bijieensis* (bootstrap 51) and was subsequently grouped with *P. lyxosi* and *P. arainonis* in a strongly supported cluster (bootstrap 87). These results confirm that both isolates belong to the genus *Pseudomonas*, although the precise phylogenetic placement of Strain5 within the genus may require further multilocus sequence or genomic analysis. However, given that only partial 16S rRNA gene sequences were analyzed and several bootstrap values were moderate, species-level identification cannot be conclusively established. Therefore, the isolates are referred to as *Pseudomonas* sp. strain 5 and strain 7 throughout the manuscript.

### Germination

**Figure 2:**
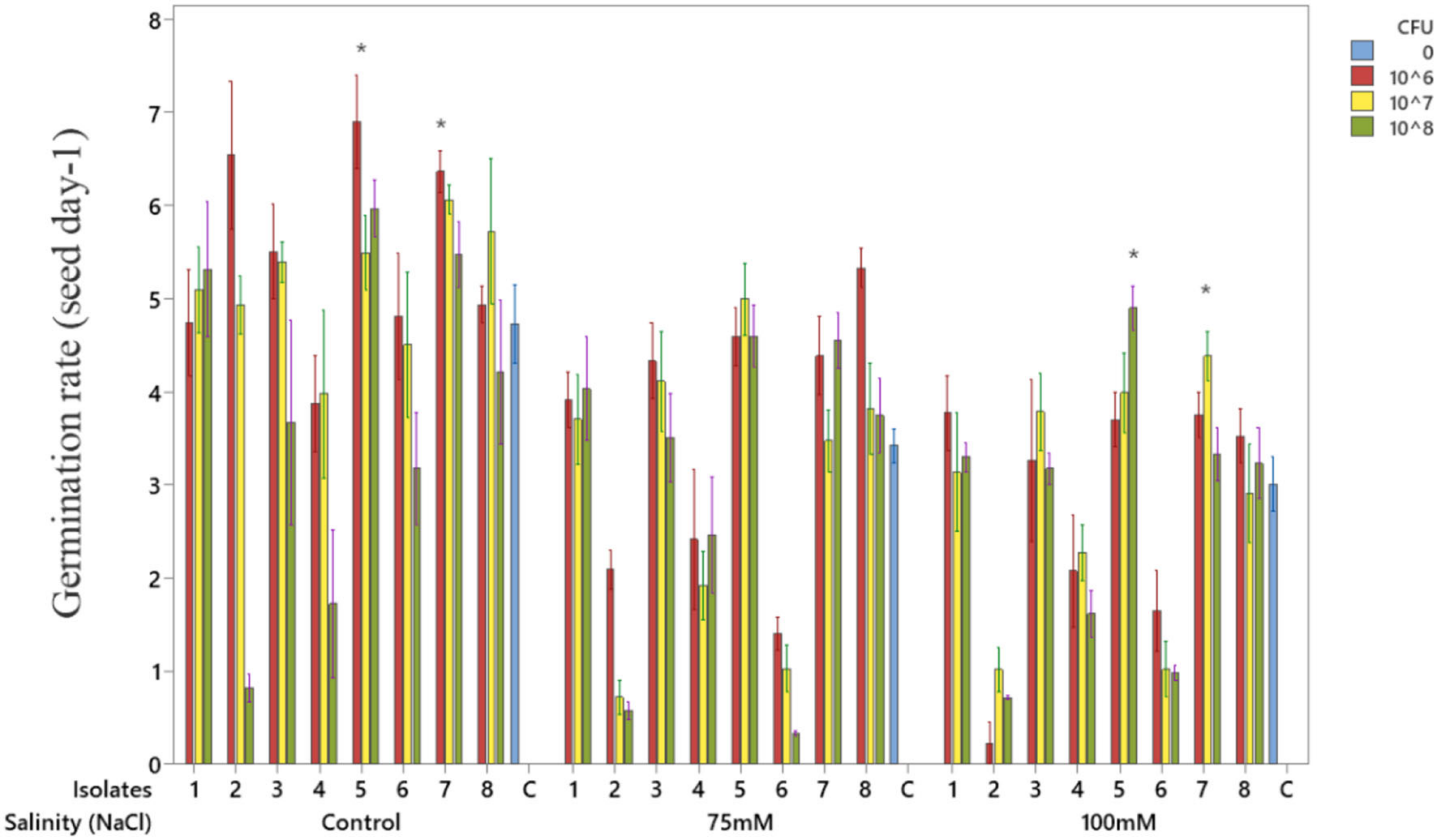
Effects of bacterial isolates on soybean seed germination rate under a range of salinity levels. Data represent the mean of 4 replicates, and error bars indicate the standard error of the mean (SEM). Asterisks (*) indicate statistically significant differences compared to the untreated control (blue column) at same salinity level (T-test, p<0.05).

The effects of bacterial isolates on soybean seed germination rate under a range of salinity levels are presented in **Figure 2**. Overall, inoculation with bacterial strains improved germination rate compared with the untreated control, although the magnitude of the effect varied among isolates, inoculum concentrations, and salinity levels. Under non-saline conditions (control), several isolates enhanced germination rate compared with the untreated control. In particular, isolates 5 and 7 showed significant improvement (p < 0.05), especially at 10^6^ CFU mL^-^¹, where germination rate reached the highest values (above 7 seeds day^-^¹). At 75 mM NaCl, germination rates decreased across all treatments compared with the non-saline control. However, inoculated seeds still performed better than uninoculated ones, with isolates 5 and 7 again maintaining relatively higher germination rates, though differences were not statistically significant. At 100 mM NaCl, germination rates declined markedly for the control treatment, reflecting the severity of salt stress. Nevertheless, inoculation with isolates 5 and 7, particularly at higher inoculum levels (10^7^–10^8^ CFU mL^-^¹), conferred a statistically significant advantage over the control (p < 0.05). Taken together, these results indicate that isolates 5 and 7 consistently enhanced seed germination under both non-saline and saline conditions, underscoring their potential as salt-tolerant, plant growth–promoting bacteria and justifying their selection for subsequent greenhouse evaluation.

#### Germination percentage

**Figure 3:**
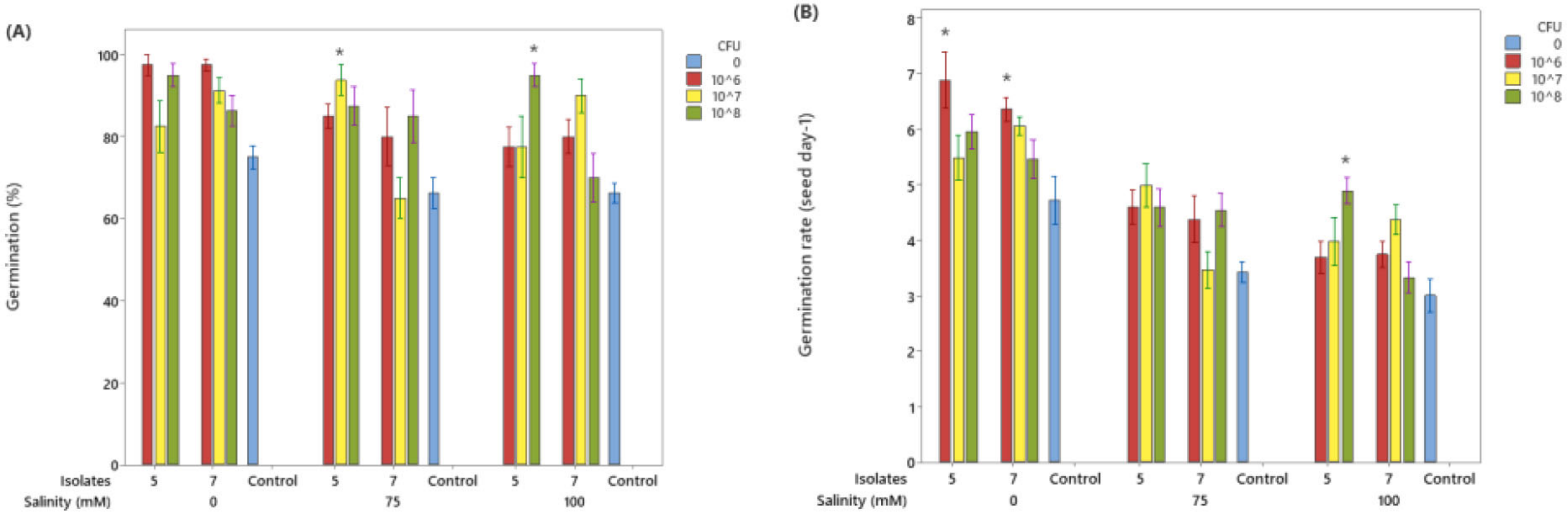
Effects of rhizobacterial isolates on soybean seed germination percentage **(A)** and germination rate **(B)** under a range of salinity levels. Data represent the mean of 4 replicates, and error bars indicate the standard error of the mean (SEM). Asterisks (*) indicate statistically significant differences compared to the untreated control (blue column) at the same salinity level (Tukey, p<0.05).

Under non-saline conditions (0 mM NaCl), seeds inoculated with isolates 5 and 7 achieved nearly complete germination (~100%), slightly higher than the untreated control. However, these differences were not statistically significant compared with the control. At 75 mM NaCl, salinity stress reduced germination overall; however, isolate 5 produced a statistically significant increase in germination percentage compared with the untreated control (Tukey, p < 0.05), while isolate 7 also improved germination numerically, but the difference was not statistically significant. At 100 mM NaCl, germination declined further, reflecting stronger salt stress. Even so, isolate 5 sustained germinations above 70% and produced a statistically significant improvement over the control (Tukey, p < 0.05), whereas isolate 7 showed a positive but non-significant effect (**Figure 3A**).

#### Germination rate

Under non-saline conditions (0 mM NaCl), inoculation with isolates 5 and 7 increased germination rate relative to the untreated control, and at 10^6^ CFU mL^-^¹ the increase was statistically significant for both isolates (Tukey, p < 0.05). At 75 mM NaCl, both isolates produced higher germination rates than the control; however, these differences were not statistically significant. At 100 mM NaCl, germination rate decreased across all treatments. Nevertheless, isolate 5 maintained a statistically significant advantage at higher inoculum levels (10^8^ CFU mL^-^¹) compared with the control (Tukey, p < 0.05), while isolate 7 showed an improvement that was not significant (**Figure 3B**).

**Figure 4:**
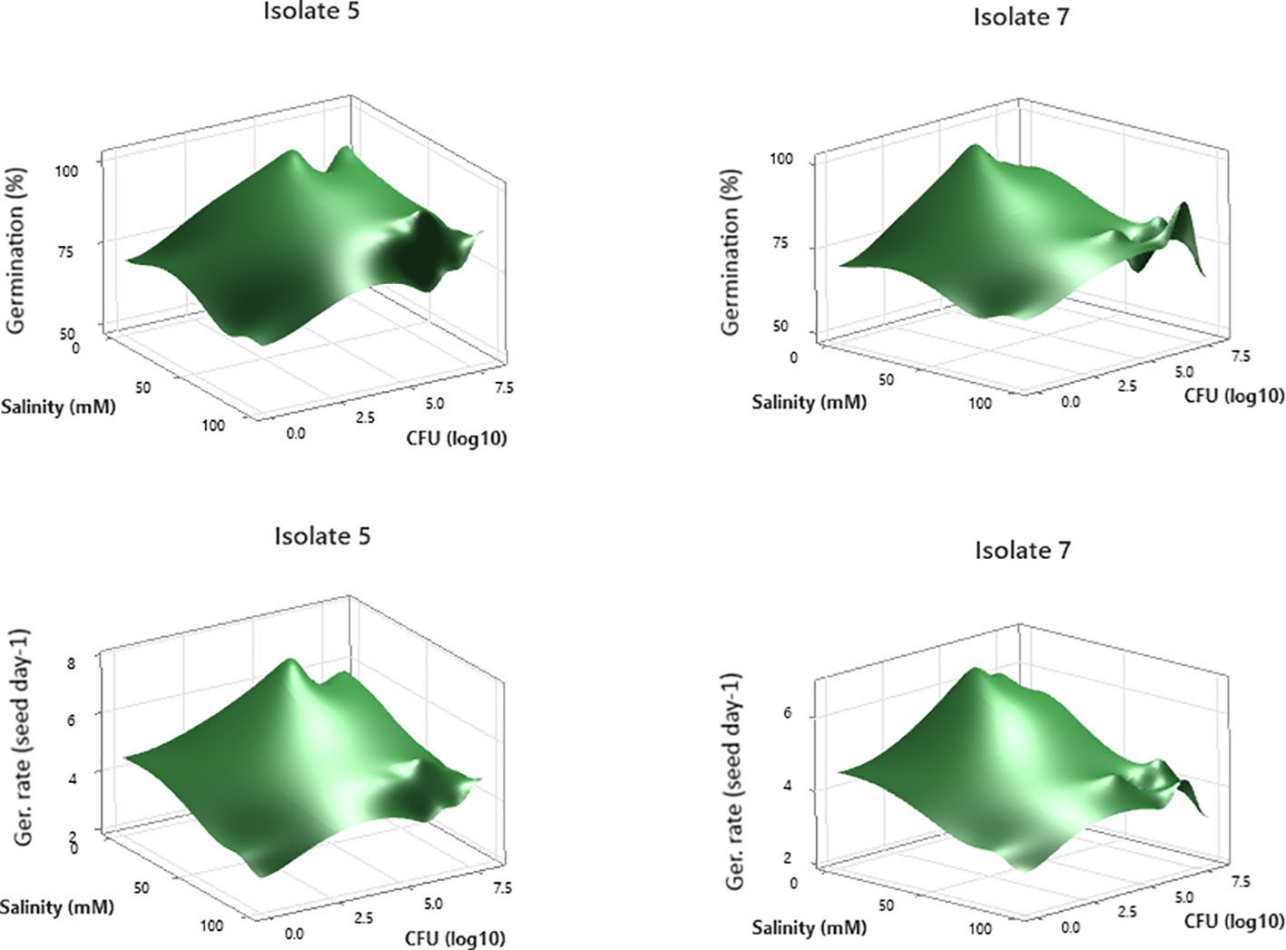
Response surface plots illustrating soybean seed germination response to bacterial concentration under varying salinity levels.

Response surface plots (**Figure 4**) showed that seed germination (%) generally increased with moderate CFU concentrations and decreased at very low or very high CFU levels. Increasing salinity negatively affected germination for seeds treated with either isolate, but the reduction was less pronounced at intermediate CFU levels, suggesting a protective effect of bacterial inoculation under salinity stress. Isolate 5 showed slightly higher germination percentages overall compared to isolate 7, especially under higher salinity conditions (**Figure 4**). Germination rate (seeds day^-^¹) followed trend similar to germination percentage. Both isolates exhibited higher germination rates at moderate CFU levels, with rates declining at very low or very high bacterial concentrations. Salinity stress reduced germination rate for both isolates, but moderate bacterial inoculation mitigated the negative impact, particularly for Isolate 5. Isolate 5 appears more effective than Isolate 7 in maintaining both germination percentage and germination rate under higher salinity and CFU variations (**Figure 4**).

### Results for greenhouse experiments

Plant height

**Figure 5:**
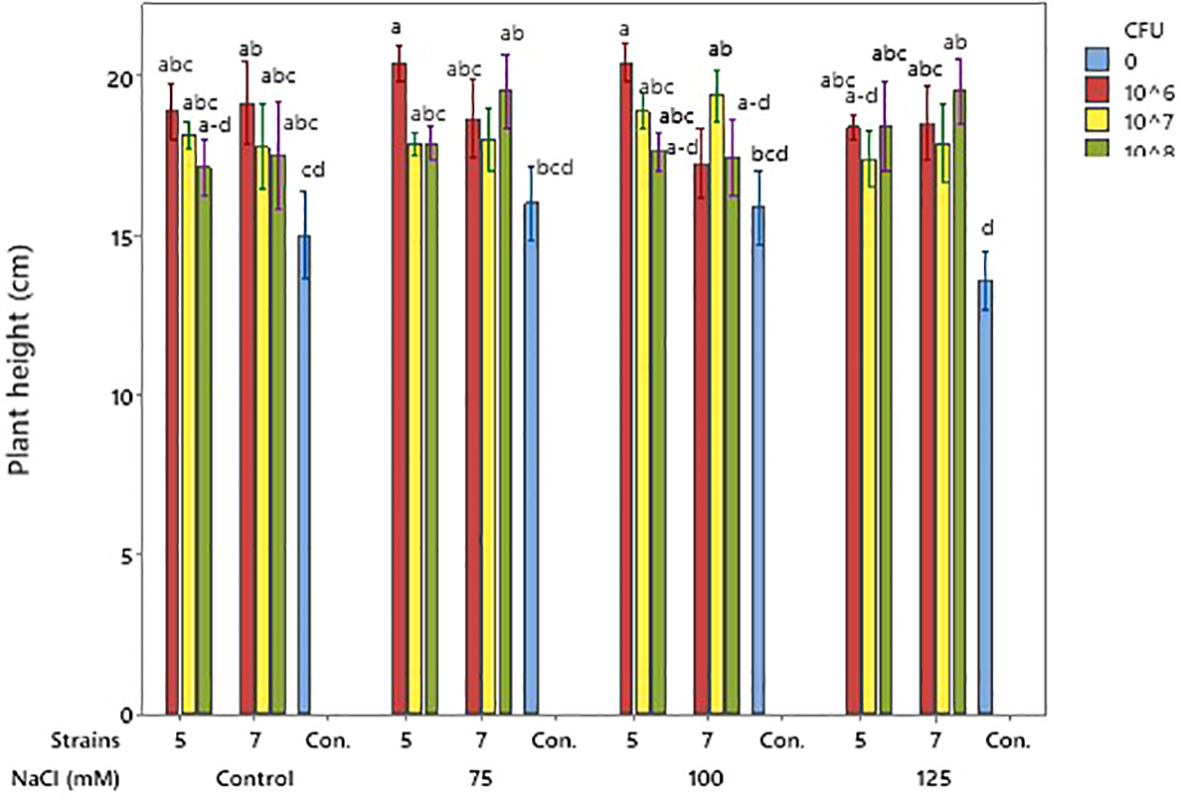
Effects of bacterial isolates on soybean plant height under pulse salinity stress. Means (n = 8) with at least one common letter are not significantly different at p < 0.05 (Tukey test). Error bars indicate SE.

Application of both bacterial isolates increased plant height compared with the non-inoculated control, across all NaCl levels. Although plant height generally declined with highest level of salinity, inoculated plants consistently maintained greater height than the control, indicating that both isolates alleviated the negative effects of salt stress. No significant difference was observed between the two isolates, suggesting that both were similarly effective in promoting growth under normal and saline conditions at the applied bacterial concentration (**Figure 5**). For isolate 5, 10^6^ CFU was most effective, however, for isolate 7, 10^6^ worked well just until 100 mM NaCl and 10^8^ CFU showed superior at the 125 mM.

### SPAD number (chlorophyll index)

**Figure 6:**
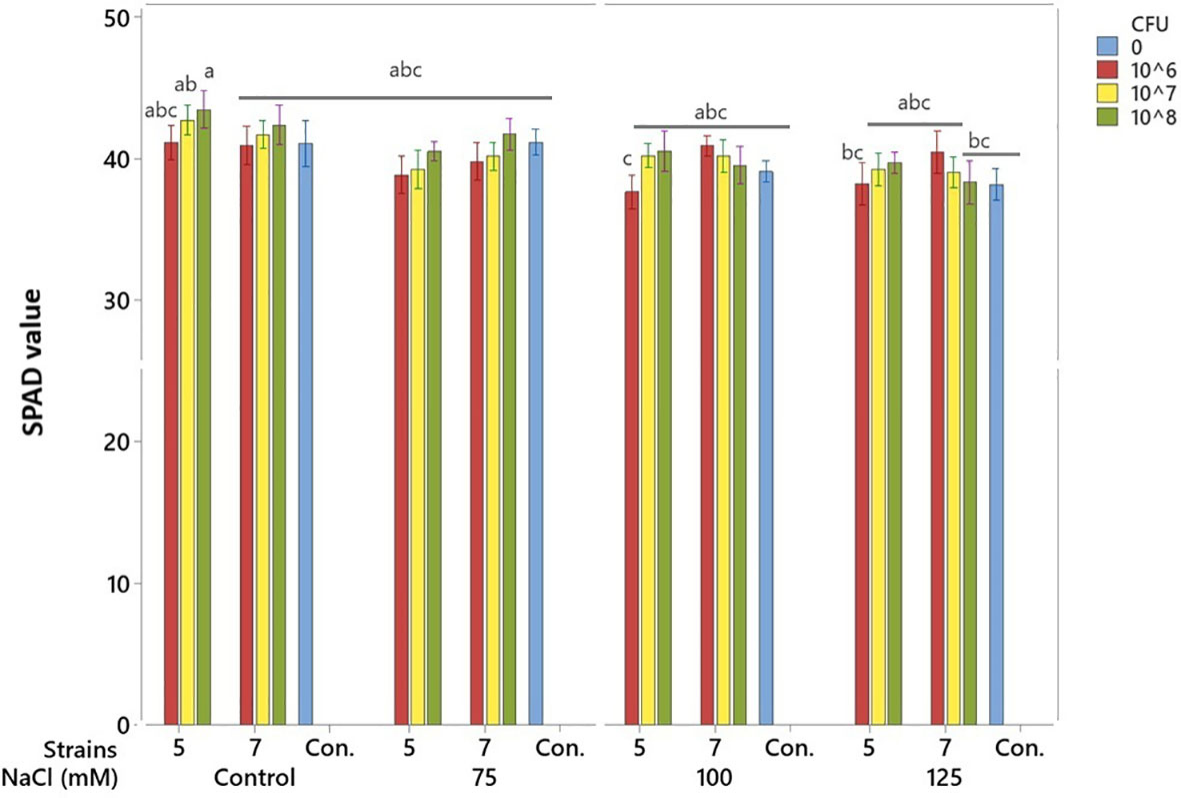
Effects of bacterial isolates on soybean SPAD values under pulse salinity stress. Means (n = 8) with at least one common letter are not significantly different at p < 0.05 (Tukey test). Error bars indicate SE.

Salinity decreased SPAD number, particularly at 125 mM. Application of bacterial isolates numerically increased SPAD number, but these increases were not statistically significant (**Figure 6**).

### Shoot dry weight and leaf area

**Figure 7:**
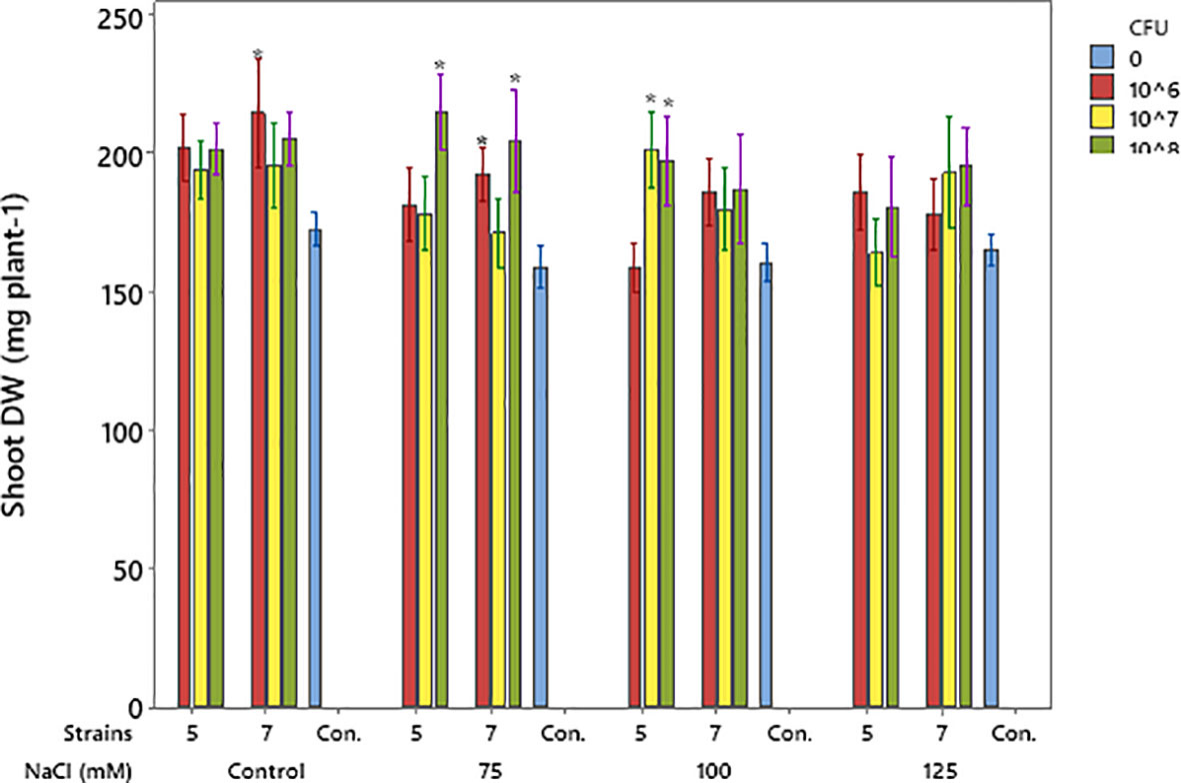
Effects of bacterial isolates on soybean shoot DW under pulse salinity stress. Means (n = 8) with a *are significantly different from their corresponding control (CFU = 0) at the same salinity level at p < 0.05 (Tukey test). Error bars indicate SE.

Salinity, at 125 mM, reduced shoot DW compared to the control. Both bacterial isolates enhanced shoot DW at salinity levels below 125 mM (**Figure 6**). Isolate 5 exerted positive effects at 75 and 100 mM, whereas Isolate 7 increased shoot dry weight across treatments from the control up to 100 mM (**Figure 7**). Application of bacterial strains improved the leaf area too. Strain 7 has a partial better performance than strain 5 regarding this trait (**Table 2**).

**Table 2:** Effects of bacterial isolates on soybean traits under salinity stress conditions.

Isolate	Leaf area (cm^2^ plant^-1^)	Shoot dry weight (mg plant^-1^)	Root length (cm plant^-1^)		Root volume (cm^3^)
			EXP 1	EXP 2	EXP2
5	35.54 a	188.32 a	32.53 a*	33.66 ab	0.908 a
7	38.89 a	191.94 a	34.62 a	33.90 a	0.905 a
Control	29.28 b	164.25 b	21.62 b	29.50 b	0.768 b

*Means that do not share a letter are significantly different according to a Tukey test (p<0.05).

### Root length, volume, and tips

**Figure 8:**
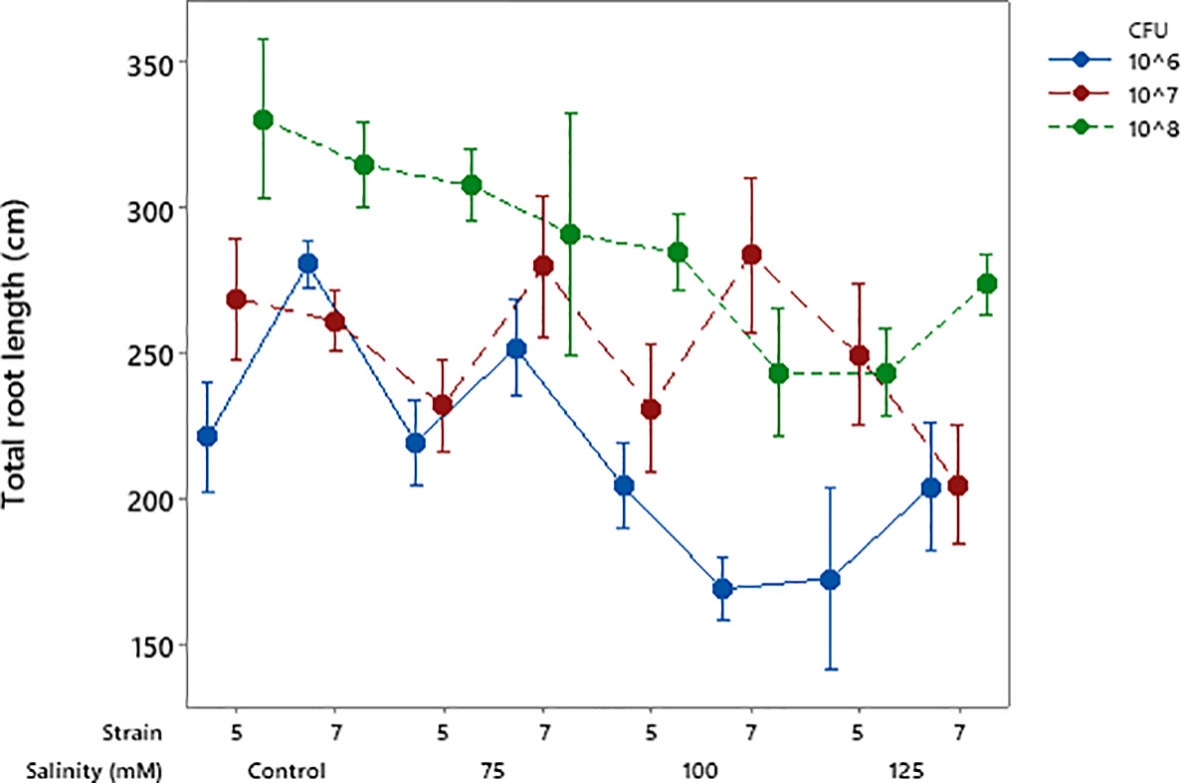
Interaction of salinity × bacterial strain × CFU on total root length of soybean. Error bars indicate SE, and means are based on four replications.

Root length data were analyzed separately due to non-homogeneity of variances. Results from both experiments showed that the bacterial strains increased root length; however, the increase was not significant in the second experiment (**Table 2**). The general trend indicated that strain number 7 performed relatively better in this regard and a CFU of 10^8^ was most effective (**Figure 8**). Root volume was not affected by salinity. However, bacterial strain increased it particularly at CFU = 10^8^.

Number of root tips

**Figure 9:**
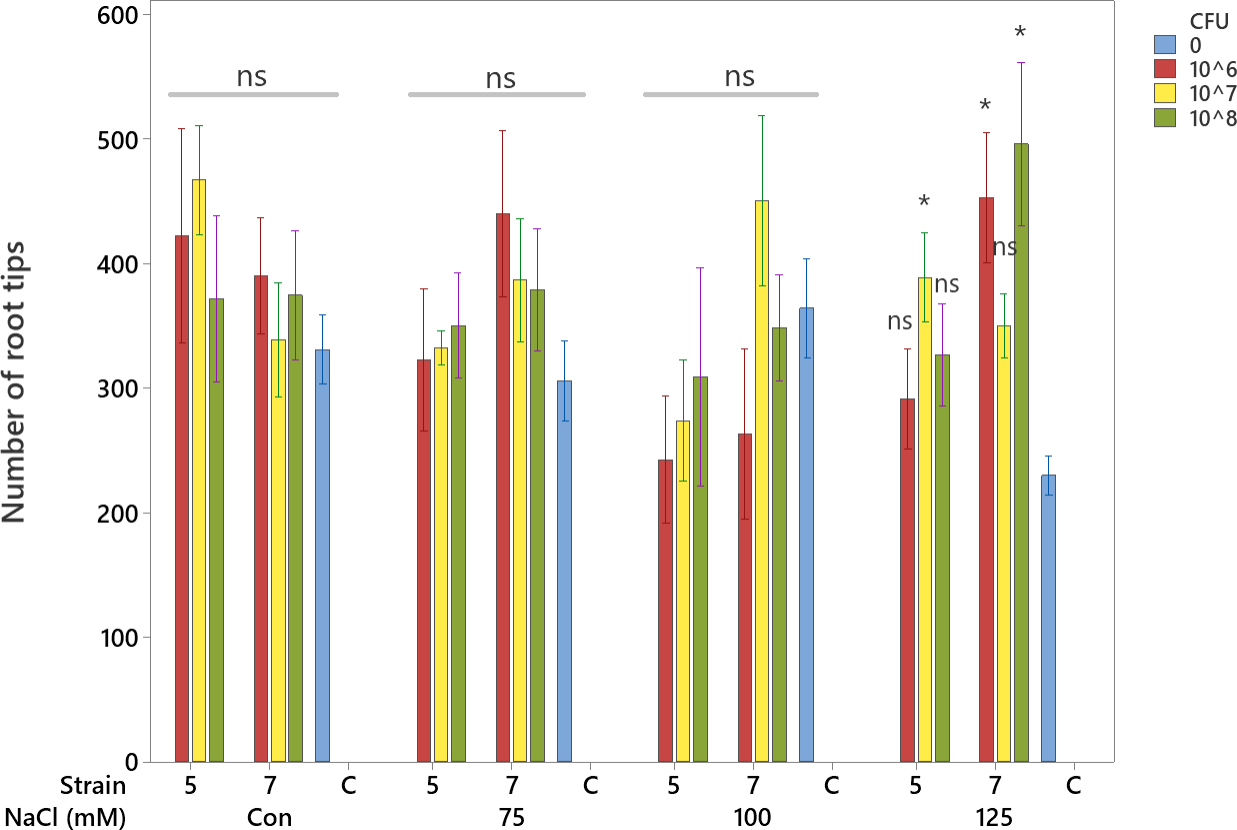
Interaction of salinity × bacterial strain × CFU on number of root tips of soybean. Error bars indicate SE, and means are based on four replications. Means with a *are significantly different from their corresponding control (CFU = 0) at the same salinity level at p < 0.05 (Tukey test).

The number of root tips was not significantly affected by bacterial inoculation under control (Con), 75 mM, or 100 mM NaCl conditions (ns). However, at 125 mM NaCl, inoculation with both bacterial strains (5 and 7) significantly increased the number of root tips compared to the uninoculated control (**Figure 9**, p < 0.05).

### Results for bacterial plant growth-promoting traits

Both strains produced auxin and strain 7 was a better auxin producer than strain 5. In addition, both strains were able to solubilize phosphate and produce siderophores. Although these strains turn the color of the NFb medium to blue in the NFb test, no visible pellicle formation was detected **Table 3**. Furthermore, neither strain exhibited growth on Ashby’s nitrogen-free medium. Therefore, the available evidence does not support the conclusion that these isolates are active nitrogen-fixing bacteria, and nitrogen fixation is unlikely to have contributed to the observed plant growth promotion.

**Table 3:** Auxin production, phosphate solubilization index (PSI), siderophore production, and nitrogen fixation ability of strains.

Isolates	Auxin (µg ml^-1^)	PSI	Siderophore production	NBF ability
5	30.20 b	2.733 a	+	–
7	36.50 a	2.900 a	+	–

Means that do not share a letter are significantly different (Tukey p=0.05). + and – represent positive and negative results in a test, respectively.

### Salinity tolerance of bacterial isolates

**Figure 10:**
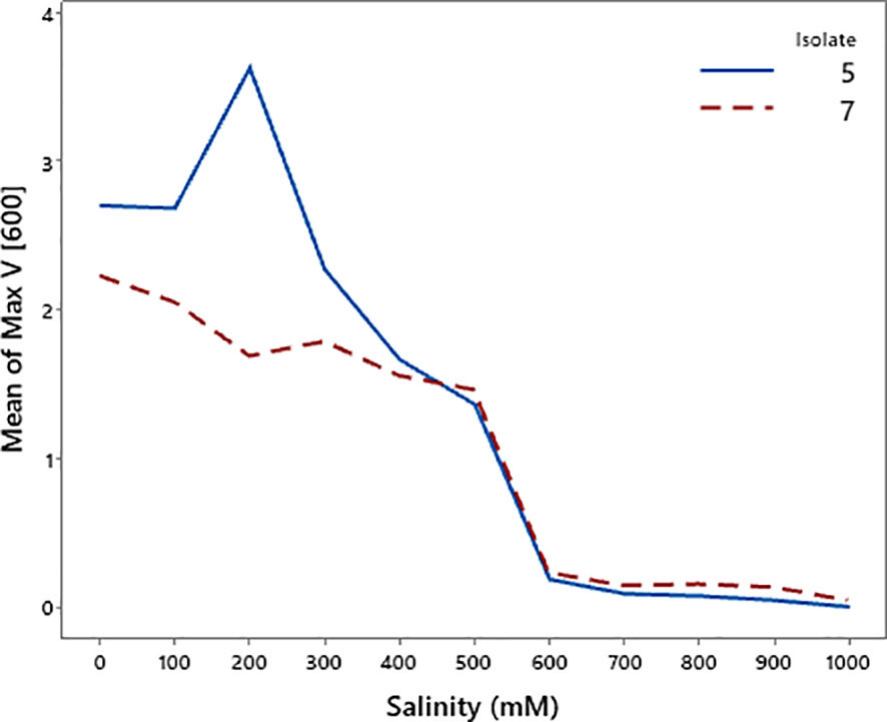
Changes in maximum growth (Max V [600]) of bacterial isolates 5 and 7 under varying salinity levels.

**Figure 11:**
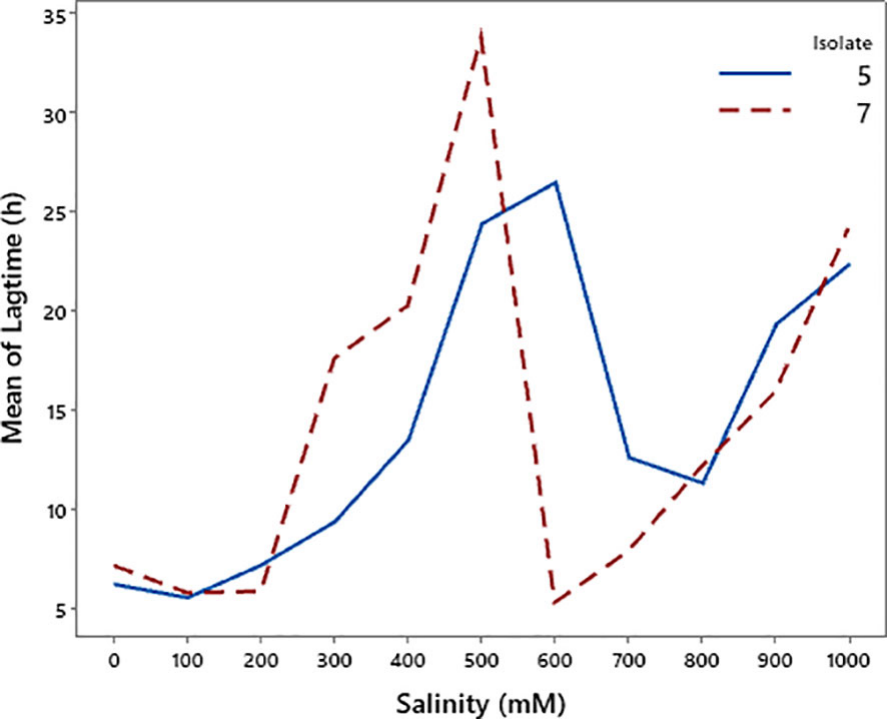
Changes in lag time of bacterial isolates 5 and 7 under varying salinity levels.

The growth response of the two bacterial isolates differed under increasing salinity levels (**Figure 10** and **11**). Isolate 5 exhibited higher maximum growth at low salinity, reaching its peak at 200 mM, but its growth declined sharply as salinity increased. In contrast, isolate 7 showed lower maximum growth overall but maintained a more gradual decrease with increasing salinity. Lag time analysis revealed that isolate 5 initiated growth more rapidly under low-salinity conditions, whereas isolate 7 displayed consistently longer lag times. Both isolates experienced a pronounced delay in growth initiation at intermediate salinity levels (400–600 mM), followed by a partial recovery at 600 mM, and then a subsequent increase in lag time at higher salinity levels. These results suggest that isolate 5 performs better under non-saline conditions, while isolate 7 exhibits relatively greater tolerance to elevated salinity.

### Growth curves of bacterial isolates under a range of salinity

**Figure 12:**
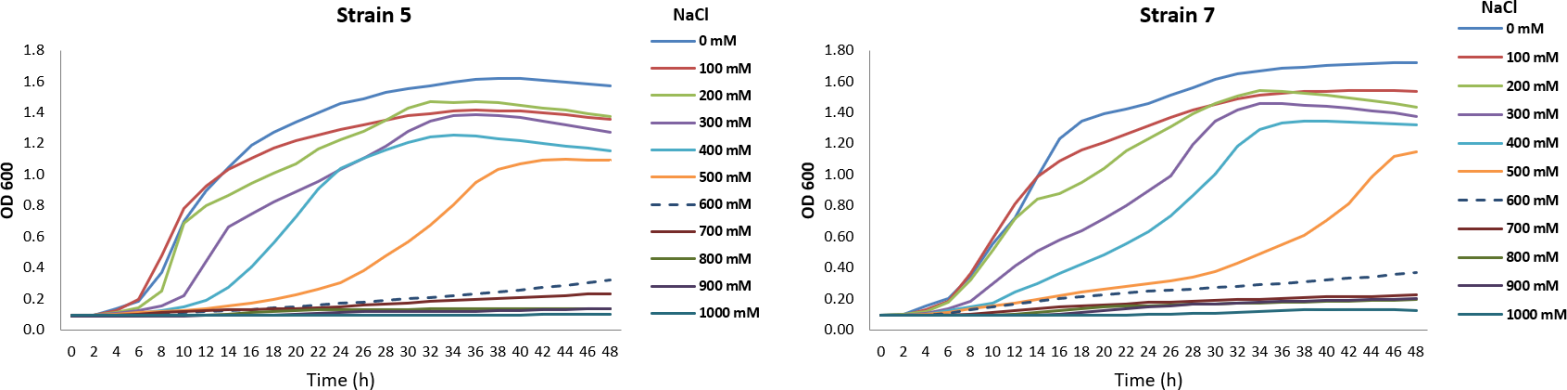
Growth curves of bacterial isolates 5 and 7 under different NaCl concentrations over 48 hours. Each observation is based on four replicates.

Optical density (OD_600_) values were recorded at regular intervals to monitor bacterial growth under increasing salinity levels (0–1000 mM NaCl). Both isolates exhibited a gradual reduction in growth rate and maximum OD_600_ with increasing NaCl concentration. Growth was only slightly affected up to 300 mM NaCl, while concentrations above 400 mM markedly inhibited cell proliferation. No substantial growth occurred beyond 700 mM for either isolate. However, Isolate 7 showed higher salt tolerance than *Isolate 5*, maintaining comparatively greater OD_600_ values under moderate to high salinity levels. Notably, at 500 mM NaCl, Isolate 7 continued to increase in optical density throughout the incubation period and did not reach a stationary phase (“level off”), suggesting a greater adaptive response to osmotic stress (**Figure 12**).

## Discussion

Plant growth-promoting rhizobacteria (PGPR) offer a sustainable and eco-friendly approach of improving crop productivity. They are known to enhance plant growth and stress tolerance, potentially through modulation of physiological processes, improved nutrient uptake, and regulation of phytohormone balance. As such, PGPR hold great promise for advancing resilient and sustainable agricultural systems (Monjezi et al., 2025; Lyu et al., 2023). Wild plants that thrive in their natural habitats without human intervention represent a valuable reservoir of rhizobacterial diversity. These microorganisms have undergone natural selection and adaptation to fluctuating environmental and climatic conditions, resulting in enhanced resilience and stress tolerance. Consequently, such naturally evolved and robust microbial strains can be considered superior candidates for mitigating the adverse effects of climate change, as they not only survive under extreme conditions but also confer stress tolerance to their associated host plants (Barnes et al., 2024).

Based on the results, bacterial strains isolated from *Poa pratensis* had a clear positive effect on soybean seed germination under both non-saline and saline conditions, indicating the potential of these isolates to enhance early plant establishment. The extent of improvement varied depending on the isolate, inoculum density, and salinity level, suggesting that the interaction between microbial activity and environmental stress plays a key role in determining germination outcomes (Bastías et al., 2022). Plants are very sensitive to salt during germination, and early salt exposure can slow seedling growth later, even in good growing conditions (Monjezi et al., 2023). Additionally, salt stress can mimic many effects of climate-related stresses such as drought and high temperature, since plants often show similar physiological responses, like reduced water uptake, oxidative stress, and hormonal imbalance. Therefore, salt stress can serve as a useful tool and indicator for selecting effective strains as plant growth promoting rhizobacteria (PGPR) under stress conditions (Zhu, 2016; Monjezi et al., 2025).

Among the eight isolates evaluated, strains 5 and 7 consistently promoted germination, particularly at moderate inoculum concentrations (10^6^–10^7^ CFU mL^-^¹), indicating an optimal range for beneficial plant–microbe interaction (**Figure 1**). The response surface analysis (**Figure 4**) revealed a clear optimum CFU range for seed germination, with reduced performance observed at the highest inoculum density. This dose-dependent pattern suggests that while moderate bacterial populations promote germination, excessive inoculum levels may negatively affect early seedling development. Such density-dependent responses have been reported in PGPR studies, where beneficial effects are strongly influenced by inoculum concentration (Lugtenberg and Kamilova, 2009). One possible explanation is the overproduction of phytohormones such as indole-3-acetic acid (IAA) at high bacterial densities. Although IAA production is a well-known mechanism underlying PGPR-mediated growth promotion, excessive levels may disrupt hormonal balance and impair root and seedling development (Spaepen et al., 2007). In addition, high microbial populations may increase metabolic competition for oxygen or produce secondary metabolites that interfere with normal seed physiology. These findings highlight the importance of optimizing inoculum concentration to maximize beneficial plant–microbe interactions while avoiding potential negative effects.

While isolates 5 and 7 performed well under non-saline conditions, isolate 5 showed stronger effects at 75 and 100 mM NaCl, maintaining over 70% germination and a higher germination rate, making it the more effective strain under salinity (**Figure 2a, b**).

Both isolates 5 and 7 were identified as belonging to the genus *Pseudomonas*, based on 16S rRNA sequencing (**Figure 1**). Previous studies have demonstrated that *Pseudomonas* sp*ecies* can colonize soybean tissues as endophytic bacteria and contribute to plant growth promotion and stress tolerance (Dubey et al., 2021; Kim et al., 2022). Also, many studies have reported that *Pseudomonas* species can enhance soybean germination under stress, which supports the present findings and suggests that the beneficial effects observed here are linked to their known plant growth, promoting traits (Costa-Gutierrez et al., 2020; Yasmin et al., 2020; Costa-Gutierrez et al., 2021; Kasotia et al., 2012). Following this, strains 5 and 7, which demonstrated statistically significant improvements in soybean germination, were selected for subsequent greenhouse evaluations. These trials aimed to assess their effectiveness in promoting early soybean growth and to confirm whether the beneficial effects observed at the germination stage persist under more complex environmental conditions. Such stepwise validation under controlled greenhouse settings provides a critical bridge between laboratory screening and greenhouse application, ensuring that selected strains possess consistent plant growth promoting activity across developmental stages and environmental gradients.

Both isolates (5 and 7) characterized in this study exhibited key plant growth promoting traits, including indole 3 acetic acid (IAA) production and phosphate solubilization. Notably, strain 7 produced higher levels of auxin than strain 5, consistent with the widespread occurrence of IAA synthesis among Pseudomonas spp.; previous surveys reported that a large proportion of *Pseudomonas* isolates produce significant amounts of IAA, which are known to modulate root architecture and may enhance nutrient absorption by influencing cell division, elongation, and differentiation in plant roots (Ferjani et al., 2019). Phosphate solubilization, another prominent feature of many Pseudomonas species, enhances phosphorus availability by releasing organic acids that convert insoluble phosphates into plant accessible forms, thereby potentially improving plant phosphorus nutrition and growth (Jo et al., 2019). Moreover, several reports have documented that isolates capable of both IAA production and phosphate solubilization frequently contribute to improved plant performance under controlled and field conditions, underlining the synergistic impact of these traits on plant nutrient acquisition (Wang et al., 2022). In the present work, both strains also produced siderophores, which may further facilitate iron uptake and indirectly support plant metabolism, although their contribution relative to IAA and phosphate solubilization requires further investigation. Importantly, despite turning the NFb medium blue in the NFb assay, neither strain grew in nitrogen free Ashby’s medium, indicating that nitrogen fixation likely does not contribute to their plant growth promoting effects under the conditions tested. Together, these findings align with extensive literature on Pseudomonas obtained from rhizosphere and endophytic niches, reinforcing IAA synthesis and phosphate solubilization as putative mechanisms through which *Pseudomonas* isolates may enhance plant growth and nutrient uptake (Ferjani et al., 2019).

Both isolates improved soybean height under salt stress, showing their ability to reduce salinity effects. Isolate 5 was most effective at 10^6^ CFU mL^-^¹, while isolate 7 required 10^8^ CFU mL^-^¹ at higher salinity (**Figure 4**). Similar effects of *Pseudomonas* strains on plant growth under stress have been reported (Costa-Gutierrez et al., 2021; Abulfaraj and Jalal, 2021). Likewise, salinity at 125 mM reduced shoot dry weight compared to the control, but both isolates enhanced shoot biomass at lower salinity levels. Isolate 5 increased shoot dry weight at 75 and 100 mM NaCl, while isolate 7 showed positive effects from non-saline to 100 mM conditions (**Figure 6**). Similar findings were reported by Dong et al. (2024), who demonstrated that a salt-tolerant *Pseudomonas aeruginosa* strain significantly enhanced plant height, stem diameter, and both fresh and dry biomass under saline conditions possibly through mechanisms such as regulation of ion homeostasis and stress-related signaling pathways, as reported in previous studies. Similarly, Abulfaraj and Jalal (2021) reported increased shoot fresh weight in soybean under stressful salinity levels when three *Pseudomonas* isolates were applied, supporting the present findings on the positive role of *Pseudomonas* species in mitigating salt-induced growth reduction. Both strains also improved leaf area, with isolate 7 performing slightly better for this trait, further confirming their growth-promoting potential under salt stress (**Table 2**). Similar findings were reported by Yasmin et al. (2020), who showed that inoculation with halotolerant *Pseudomonas alcaligenes* improved soybean growth and leaf area under salinity. Likewise, Win et al. (2023) found that co-inoculation with *Bradyrhizobium* and *Pseudomonas* enhanced soybean biomass and leaf area recovery, confirming the role of *Pseudomonas* in mitigating salt stress and promoting growth. Both isolates improved root length and volume, with strain 7 performing better at 10^8^ CFU mL^-^¹. Although the increase in root length was not always significant, the trend showed better root growth under salt stress (**Table 2**). Similar to our results, earlier studies reported that *Pseudomonas* can improve soybean root growth under salinity by increasing root length and structure (Kasotia et al., 2016; Egamberdieva et al., 2017) and boosting root biomass when used with other beneficial bacteria (Win et al., 2023).

Although several plant growth-promoting traits were identified *in vitro* (except for auxin quantification), the proposed mechanisms underlying growth enhancement should be considered putative. Further in planta physiological and molecular analyses are required to confirm the exact pathways involved.

## Conclusions

The isolation, identification, and formulation of rhizosphere bacteria from stress-tolerant plants represents a promising approach for developing new biofertilizers that support sustainable agriculture and enhance resilience to climate change. In this study, two root endophyte bacterial isolates obtained from *Poa pratensis* exhibited notable tolerance to salinity stress (up to 500 mM NaCl) and demonstrated key plant growth-promoting traits, including indole-3-acetic acid (IAA) production, phosphate solubilization, and siderophore production. These characteristics suggest their potential as biofertilizer candidates that may improve plant performance under saline conditions. However, further research is required to evaluate their efficacy under field and soil environments and to elucidate the underlying mechanisms involved in their salt tolerance and plant growth-promoting activities. Understanding these mechanisms at physiological and molecular levels will be essential for optimizing their application and ensuring their stability and effectiveness in sustainable crop production systems.

## Data Availability

The raw data supporting the findings of this study are deposited in the Zenodo repository, https://doi.org/10.5281/zenodo.19001612.
